# Phylloxerids share ancestral carotenoid biosynthesis genes of fungal origin with aphids and adelgids

**DOI:** 10.1371/journal.pone.0185484

**Published:** 2017-10-11

**Authors:** Chaoyang Zhao, Paul D. Nabity

**Affiliations:** Department of Botany and Plant Science, University of California, Riverside, California, United States of America; Natural Resources Canada, CANADA

## Abstract

Gene transfer among reproductively isolated organisms can lead to novel phenotypes and increased fitness. Among the Sternorrhyncha, a suborder of plant sap-feeding hemipteran insects, both aphids and adelgids acquired carotenoid biosynthesis genes from a fungal donor that result in ecologically relevant pigmentation. Phylloxerids form another family that are closely related to aphids and adelgids and share similar pigmentation, but are largely uncharacterized for their presence and number of pigment genes that have duplicated among aphids. Here, we examined the transcriptomes of nine phylloxerid species, and performed PCR to amplify carotenoid genes from their genomic DNA. We identified carotenoid cyclase/synthase and desaturase genes in each species and demonstrated that they share the common fungal origin as those of aphids and adelgids based on their exon-intron gene structures and phylogenetic relationships. The phylogenetic analyses also indicated that carotenoid genes evolved following the differentiation of aphids, adelgids, and phylloxerids at the levels of family, genus, and species. Unlike aphids that duplicated these genes in their genomes, phylloxerids maintained only single copies, and some species may lack expression of certain genes. These results suggest that the phylloxerid lifestyle undergoes reduced selection pressure to expand carotenoid synthesis genes, and provides insight into these gene functions in insects.

## Introduction

In contrast with traditional reproduction where genes are transmitted vertically from a parent to offspring, horizontal or lateral gene transfer (HGT or LGT) is the transmission of genes between reproductively isolated organisms. HGT is an important factor driving the evolution of microorganisms, including the acquisition of new features like antibiotic resistance [1]. Although a great number of reported HGT events occur between microorganisms, recent studies demonstrate that insects readily incorporate foreign genes into their genomes [2]. Next-generation sequencing technologies have facilitated the discovery of microbial genes incorporated into the genomes of insect species belonging to a variety of orders including Diptera, Hymenoptera, Blattodea, Hemiptera, Coleoptera, Phasmatodea, and Lepidoptera [3–11], but the function of many of these HGTs remains unresolved.

Insect endosymbionts are potential gene donors that may integrate DNA fragments as large as their whole genome into those of their hosts [12]. Although some horizontally acquired genes undergo degradation through mutational processes such as frame-shift gene mutations and eventually become pseudogenes, many genes remain and evolve functional roles that increase the fitness of recipient organisms. Several studies indicated that the acquisition of microbial genes in insect genomes facilitates nutrient digestion and metabolism, including the catabolism of dietary carbohydrates and the biosynthesis of amino acids, vitamins, and carotenoids [2, 13–19].

Carotenoids are lipophilic pigments that play important roles in photosynthesis and cell protection because of their light energy absorbing and transferring, and anti-oxidant properties [20]. Plants, algae, and certain bacteria and fungi are the predominant carotenoid producers in nature. While animals require carotenoids for vision, communicative coloration, immune-system enhancement, and other physiological functions, members of Animalia were long believed to be incapable of synthesizing carotenoids *de novo* and thus must obtain them from diets or through symbiosis [21–22]. However, this has been challenged since the discovery of two carotenoid biosynthesis genes that encode a lycopene cyclase/phytoene synthase fusion protein and a phytoene desaturase, respectively, in the genomes of aphids, mites, and gall midges [13, 23–24]. These two proteins, composed of three enzymatic domains in total, are highly conserved in both microbes and eukaryotes, and catalyze the earliest protein transformations in the carotenoid pathway [25]. Interestingly, these arthropod-derived carotenoid genes are all of fungal origins and were gained in different HGT events given the distant phylogenetic relationships among aphids, gall midges, and mites. Unlike carotenoid genes in plants, algae, and bacteria, fungal *cyclase/synthase* and *desaturase* are typically arranged to form a tail-to-tail cluster in the genome, giving these genes a distinct structural motif; arthropod carotenoid genes share this motif, confirming their fungal origins [24].

As the first animal species reported to be capable of producing its own carotenoids, the pea aphid (*Acyrthosiphon pisum*) possesses multiple copies of both carotenoid cyclase/synthase and desaturase genes [13]. One of the desaturase genes is of ecological significance because it determines color polymorphism in aphid populations and impacts their susceptibility to natural enemies. Carotenoid genes, specifically *desaturase*, were later found to be commonly present in other aphid species, as well as their close relatives, adelgids; all have various gene copy numbers that are all closely related [26]. These patterns indicated that aphids and adelgids share the same carotenoid gene origin, which was then diversified through lineage-specific gene duplication and/or deletion. Of note, no carotenoid genes were detected from species of Aleyrodidae and Psyllidae, families more distantly related to aphids within the suborder Sternorrhyncha [26].

Phylloxerids are herbivorous hemipterans closely related to aphids and adelgids. This family (Phylloxeridae) consists of many species that are able to manipulate host physiological processes and induce plant galls [27, 28]. Given the omnipresence of carotenoid genes in aphids and adelgids but absence in whiteflies (Aleyrodidae) and psyllids, we were interested in understanding whether phylloxerids acquired fungal carotenoid genes as aphids and adelgids did. Further, we attempted to answer whether these phylloxerid genes, if present, underwent duplication as observed in many aphid species [29], given their life histories: phylloxerids feed on parenchyma whereas all other aphidomorphs feed on phloem. We hypothesize that 1) if phylloxerids possess carotenoid genes they were acquired before the separation into aphids, adelgids, and phylloxerids, and thus 2) these genes can predict the phylogenetic relationships among the Sternorrhyncha families as well as genera within Phylloxeridae.

## Materials and methods

### 1) Phylloxeridae insect genomic DNA extraction

Nine known species within Phylloxeridae were used for genomic DNA extraction. They were all collected from fields in the United States as previously described [30]. Briefly, these include eight *Phylloxera* species that form galls on *Carya* species and *Daktulosphaira vitifoliae* that galls both roots and leaves of *Vitis* species. Among the former, three (*P*. *caryaecaulis*, *P*. *subelliptica*, *P*. *caryaemagna*,) induce stem/petiole galls, three (*P*. *caryaefallax*, *P*. *foveata*, *P*. *foveola*) induce leaf galls, and one (*P*. *caryaevenae*) feeds across hosts causing crinkle/folds in leaf veins on hickories (*Carya* spp.), but one (*P*. *quercus*) lives freely on oaks (*Quercus* spp.).

Insect specimens were collected in the field and stored in 95% ethanol at -20°C, and the insect genomic DNA was extracted using the DNeasy Blood & Tissue Kit (Qiagen, USA) according to the protocol provided. We used 10–20 individuals for DNA extraction per biological replicate, and performed two replicates per insect species.

### 2) Carotenoid gene identification

Previously, we generated whole-body transcriptomes of the nine Phylloxeridae species described above [30]. To identify Phylloxeridae carotenoid genes, we first used the *Ac*. *pisum* carotenoid cyclase/synthase (XP_001943170.1) and desaturase (XP_001943225.2) protein sequences to search against these nine transcriptome databases (TBLASTN, evalue < 1×10^−5^) and retrieved the hit sequences whose coding regions were subsequently predicted using the ORF Finder tool at NCBI (https://www.ncbi.nlm.nih.gov/orffinder/). We then validated these sequences by searching (BLASTX, evalue < 1×10^−5^) them back to the NCBI non-redundant protein database and those not hitting a carotenoid cyclase/synthase or a carotenoid desaturase were removed (see S1 and S2 Files). Because RNA-seq assemblies do not contain information of unexpressed genes and comprise splicing variants and truncated transcripts that are encoded by same gene loci, we chose to perform PCR to obtain the number of carotenoid genes from the genomic DNA, which would also allow us to resolve the exon-intron structure of these genes. Degenerate PCR primers (see S3 File) were designed based on the alignments of Phylloxeridae and *Ac*. *pisum* coding sequences with avoidance of each primer crossing the exon-intron splice sites determined by the *D*. *vitifoliae* draft genome sequence on AphidBase (http://bipaa.genouest.org/is/aphidbase/daktulosphaira_vitifoliae/). For each carotenoid cyclase/synthase gene amplification, two nested PCR reactions were separately performed to yield two overlapping DNA fragments: *cs1* (cs1f1 and cs1r1 as the first-round PCR primers, and cs1f2 and cs1r2 as the second-round primers), and *cs2* (cs2f1 and cs2r1 as the first-round primers, and cs2f2 and cs2r2 as the second-round primers). For each carotenoid desaturase gene amplification, nested PCR was conducted using the first-round primers ds-f1 and ds-r1 and the second-round primers ds-f2 and ds-r2. The PCR condition is 94°C for 5 min, followed by 35 circles of 94°C for 15 sec, 52°C for 20 sec and 72°C for 2 min. The combination of degenerate primers and nested PCR increases the chance of amplifying the carotenoid genes in all Phylloxeridae species and meanwhile ensures PCR specificity. PCR products were subsequently purified using the QIAquick PCR Purification Kit (Qiagen, USA) according to the supplier’s protocol and submitted for sequencing. The resulting sequences of carotenoid *cyclase/synthase* fragments *cs-1* and *cs-2* were merged into one, and the gaps in the coding regions were filled using the transcript sequences with identities ≥ 99%, if available.

To identify all possible carotenoid genes in an Adelgidae species, we used the same *Ac*. *pisum* carotenoid protein sequences (XP_001943170.1 and XP_001943225.2) for TBLASTN searches against *Adelges tsugae* transcriptome (NCBI # PRJNA242203), the only available public adelgid transcriptome/genome database to our knowledge. The hit sequences were retrieved for further analysis.

Regarding the *Myzus persicae* carotenoid gene identification, we performed a genome-wide screening using the BLASTP searches against the *My*. *persicae* genome assembly and its predicted gene models on AphidBase (http://bipaa.genouest.org/is/aphidbase/myzus_persicae/). From the *My*. *persicae* Clone G006 Proteins OGS1.0 database, eight carotenoid cyclase/synthase and five desaturase sequences were hit, respectively. The cyclase/synthase hits are MYZPE13164_G006_v1.0_000134340 (only the last six digits are shown for simplicity, *i*.*e*., 134340, hereafter for all *My*. *persicae* proteins), 134290, 134420, 134380, 134360, 134370, 134390 and 134400, and the desaturase hits are 134410, 134480, 203460, 134430, and 134350. Among these, two cyclase/synthase hits, 134420 (1194-aa) and 134380 (1153-aa), and one desaturase hit, 134430 (725-aa), are abnormally long. Thus, we conducted manual annotation on their corresponding genomic DNA sequences based on their similarity to *Ac*. *pisum* carotenoid genes and predicted one new 134420 (599-aa), two new 134380 (namely, 134380–1 and 134380–2, 600-aa and 589-aa long, respectively), and one new 134430 (530-aa) proteins. In summary, nine cyclase/synthase (134340, 134290, 134420, 134380–1, 134380–2, 134360, 134370, 134390 and 134400) and five desaturase (134410, 134480, 203460, 134430, and 134350) proteins were identified from the *My*. *persicae* genome.

### 3) Phylogenetic analysis

Phylloxeridae carotenoid gene sequences obtained above were used to predict exon-intron splice sites based on the transcript/genomic DNA sequence alignments of the same insect species. In species that transcript sequences were not available or intact enough to resolve gene structure, we aligned its gene sequence to the transcript sequence of a closely related species, e.g., *D*. *vitifoliae*, to predict the exon-intron splice sites. The intron sequences were removed and the resulting exons were merged into the coding sequences.

All carotenoid gene coding sequences retrieved were translated into protein sequences using the DNA translation tool at http://web.expasy.org/translate/. The fungal and bacterial protein sequences were obtained from the NCBI Non-redundant protein database using BLASTP searches with the *Ac*. *pisum* carotenoid proteins as queries. Because carotenoid cyclase and synthase are encoded by different genes in bacteria, we concatenated *Salisaeta longa* cyclase (NCBI # WP_028566860) and synthase (NCBI # WP_028566847) into a single sequence, as well as to *Rhodothermaceae bacterium* cyclase (NCBI # WP_068126813) and synthase (NCBI # WP_068127706). All deduced cyclase/synthase and desaturase protein sequences (see S4 & S5 Files), including those reported previously in *Ac*. *pisum* [13], *Tetranychus urticae* [23] and *Mayetiola destructor* [24] were aligned, respectively, using MAFFT (version 7.130) with ‘auto’ setting [31]. The poorly-aligned or highly divergent regions were removed from the alignments on the Gblocks server with the less stringent options [32]. To conduct maximum likelihood phylogenetic analysis, the optimal substitution model, which was the ‘Le and Gascuel’ (LG) model [33] for both alignments incorporated a discrete gamma distribution (+G, shape parameter = 2) to model evolutionary rate differences among sites, was determined according to the Bayesian Information Criterion (BIC) using the tool ‘Find Best DNA/Protein Models (ML)’ on MEGA (v6) [34]. The phylogenetic trees, setting the bacterial sequences as outgroups, were constructed by testing 1000 replicates on MEGA.

## Results

### 1) Single copy of carotenoid genes in Phylloxeridae

**Fig 1:**
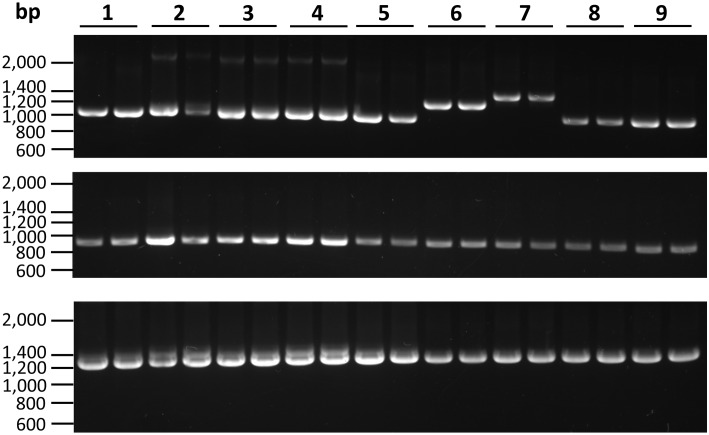
PCR amplification of carotenoid genes. The agarose gels show single copies/genes for *cyclase/synthase* fragment 1 (*cs-1*, top panel), fragment 2 (*cs-2*, middle panel), and the *carotenoid desaturase* fragment (bottom panel). The numbers 1 through 9 on the top represent species *Phylloxera caryaefallax* (1), *P*. *subelliptica* (2), P. *caryaemagna* (3), *P*. *caryaecaulis* (4), *P*. *quercus* (5), *P*. *foveata* (6), *P*. *foveola* (7), *P*. *caryaevenae* (8), and *Daktulosphaira vitifoliae* (9), respectively. Two biological replicates were used for each species. The locations of primers used for PCR are shown in Fig 2.

We used the pea aphid carotenoid cyclase/synthase (XP_001943170.1) and desaturase (XP_001943225.2) protein sequences as queries to search their homologs from the transcriptomes of nine Phylloxeridae species, including the grape phylloxera *Daktulosphaira vitifoliae* and eight *Phylloxera* spp. [30]. Transcripts of both carotenoid cyclase/synthase and desaturase genes were identified in all the nine species except no *carotenoid desaturase* was found in *P*. *foveola* (see S2 File). To validate the number of carotenoid genes in each species and investigate their exon-intron architecture, we designed degenerate primers (see S3 File) based on the coding region sequence alignments of Phylloxeridae transcripts, and conducted nested PCR using the Phylloxeridae genomic DNA extracted. Our primers were designed to amplify two overlapping *carotenoid cyclase/synthase* fragments (*cs-1* and *cs-2*), each of which crossed one of the two putative introns of the gene, respectively, and one *carotenoid desaturase* fragment that crosses the only putative intron of this gene. Of note, the number and the putative positions of *cyclase/synthase* and *desaturase* introns were predicted according to the transcript/genome DNA sequence alignments of *D*. *vitifoliae* whose genome has been sequenced. For each of the nine Phylloxeridae species, a single DNA fragment was amplified from each nested PCR, including *cs-1*, *cs-2* and the *carotenoid desaturase* fragment (Fig 1), indicating that there may be only a single copy of carotenoid genes in Phylloxeridae genomes. Sequencing these PCR products validated that each contains one DNA sequence. In addition, screening of both carotenoid genes in the *D*. *vitifoliae* genome sequence hits a single locus, respectively. Interestingly, the PCR products of carotenoid genes appeared to share similar size among all Phylloxeridae species with exception of *cs-1* amplicons in *P*. *foveata* and *P*. *foveola* that are apparently larger than those of others (Fig 1).

### 2) Conservation of exon-intron structure

**Fig 2:**
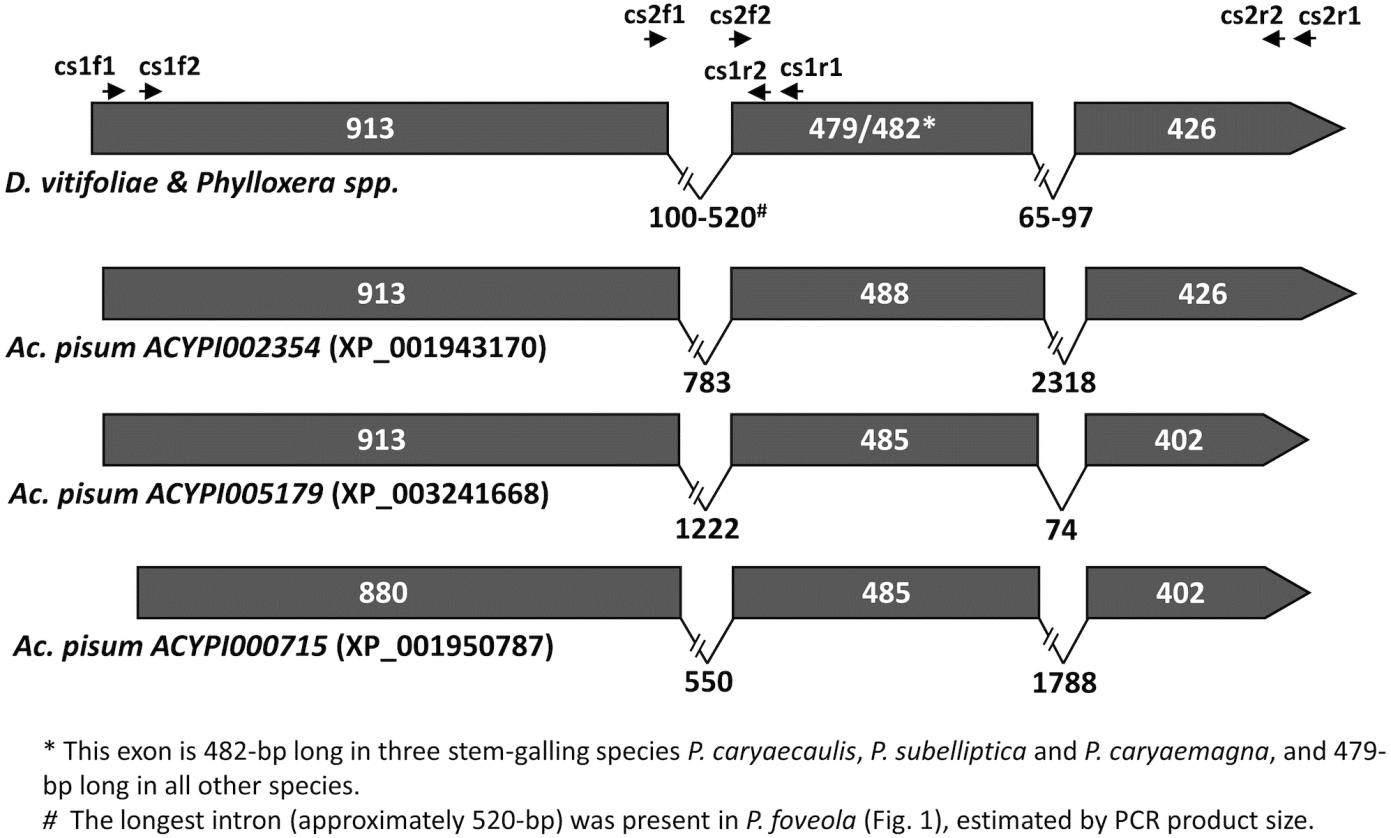
Comparison of *carotenoid cyclase/synthase* exon-intron structure in Phylloxeridae and *Acyrthosiphon pisum*. Solid boxes denote exons and lines connecting exons denote introns. Length of exons and introns is the number of base pairs indicated. Arrows on the top indicate the positions of the primers used for nested PCR of the two fragments (*cs-1* and *cs-2*) of *carotenoid cyclase/synthase*.

We determined the exon-intron junctions of Phylloxeridae carotenoid genes based on the transcript/gene sequence alignments and found that both *cyclase/synthase* and *desaturase* are highly conserved in the exon-intron gene structure in Phylloxeridae. Similar to their *Ac*. *pisum* homologs, all Phylloxeridae cyclase/synthase genes share identical exon-intron splice sites and are composed of three exons separated by two introns within the coding region (Fig 2). The first and third exons are 913-bp and 426-bp long, respectively, in all Phylloxeridae cyclase/synthase genes, as well as in *ACYPI002354* which encodes the protein XP_001943170 of *Ac*. *pisum*. In contrast, length of the second exon varies slightly which is 482-bp in three stem-gall-inducing species, *P*. *caryaecaulis*, *P*. *subelliptica*, and *P*. *caryaemagna*, and 479-bp in all other non-stem-galling Phylloxeridae. Compared to the conservation of exon sizes, however, the intron sizes of *cyclase/synthase* are divergent in Phylloxeridae, especially for the first intron that connects the first and second exons, with shortest (100-bp) in *D*. *vitifoliae* and longest (~ 520-bp) in *P*. *foveola*. (Figs 1 & 2). Unlike *Ac*. *pisum* whose *cyclase/synthase* introns can be as long as 2318-bp, the Phylloxeridae homologs comprise relatively small introns with the second one all shorter than 100-bp.

### 3) Phylogenetic relationships of Phylloxeridae carotenoid genes

**Fig 3:**
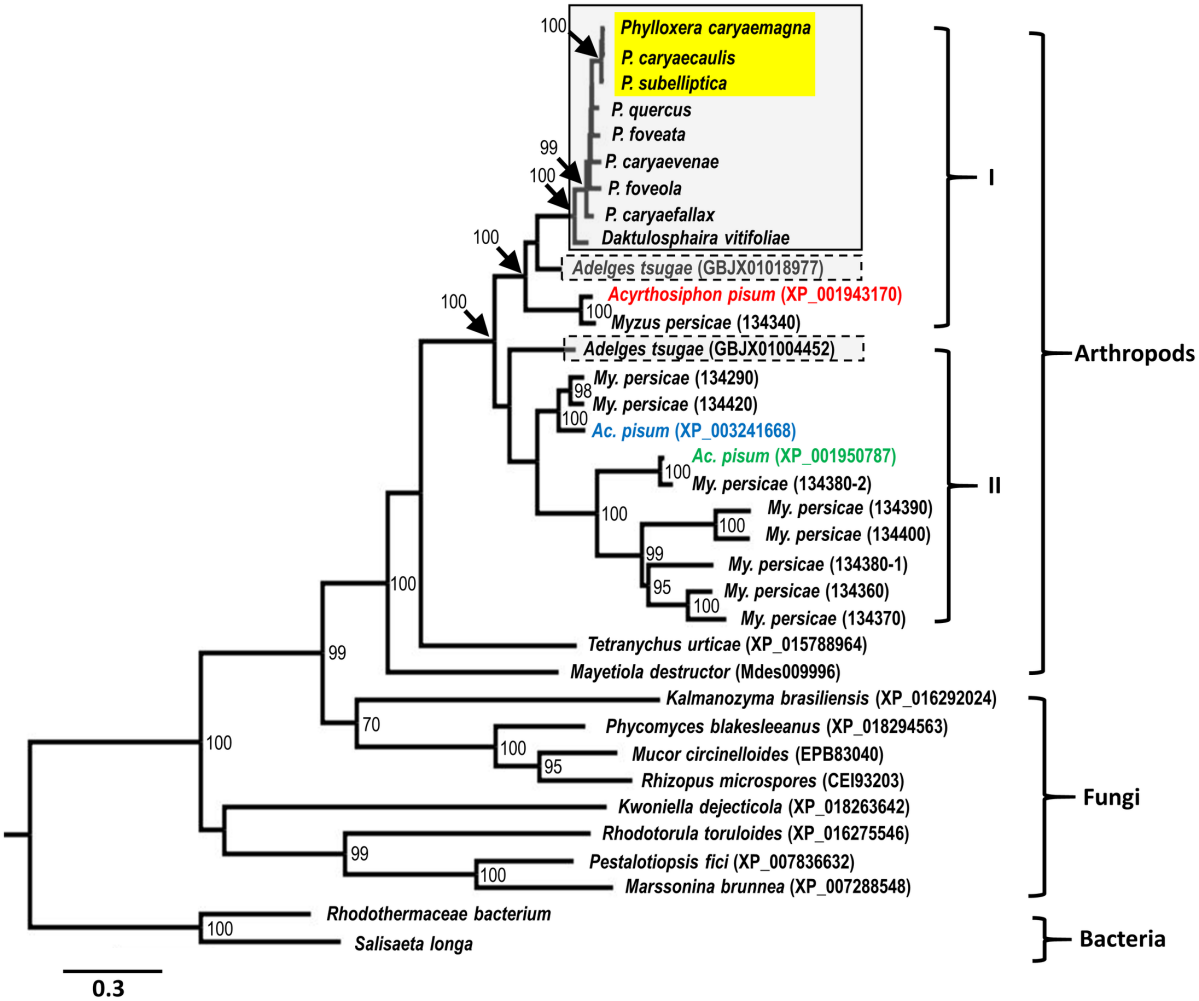
Maximum-likelihood inferred phylogeny of carotenoid cyclase/synthase proteins. Accessions of sequences retrieved from the public databases are indicated in parenthesis as Proteins OGS1.0 numbers for *My*. *persicae* (Clone G006), *Mayetiola destructor* gene model number, or NCBI number elsewhere. Phylloxeridae including *Daktulosphaira* an*d Phylloxera* species are boxed in solid lines and Adelgidae is boxed in dotted lines. Three stem gall-inducing *Phylloxera* species are highlighted in yellow. Carotenoid cyclase/synthases in red, blue and green shown here form tail-to-tail pairs, respectively, with *Ac*. *pisum* desaturase with same color shown in Fig 4, based on their gene locations in the genome. Branches with bootstrap (1000 replicates) support values ≥ 70% are labelled. The tree is rooted with bacterial homologs and is drawn to scale, with branch lengths measured in the number of substitutions per site.

**Fig 4:**
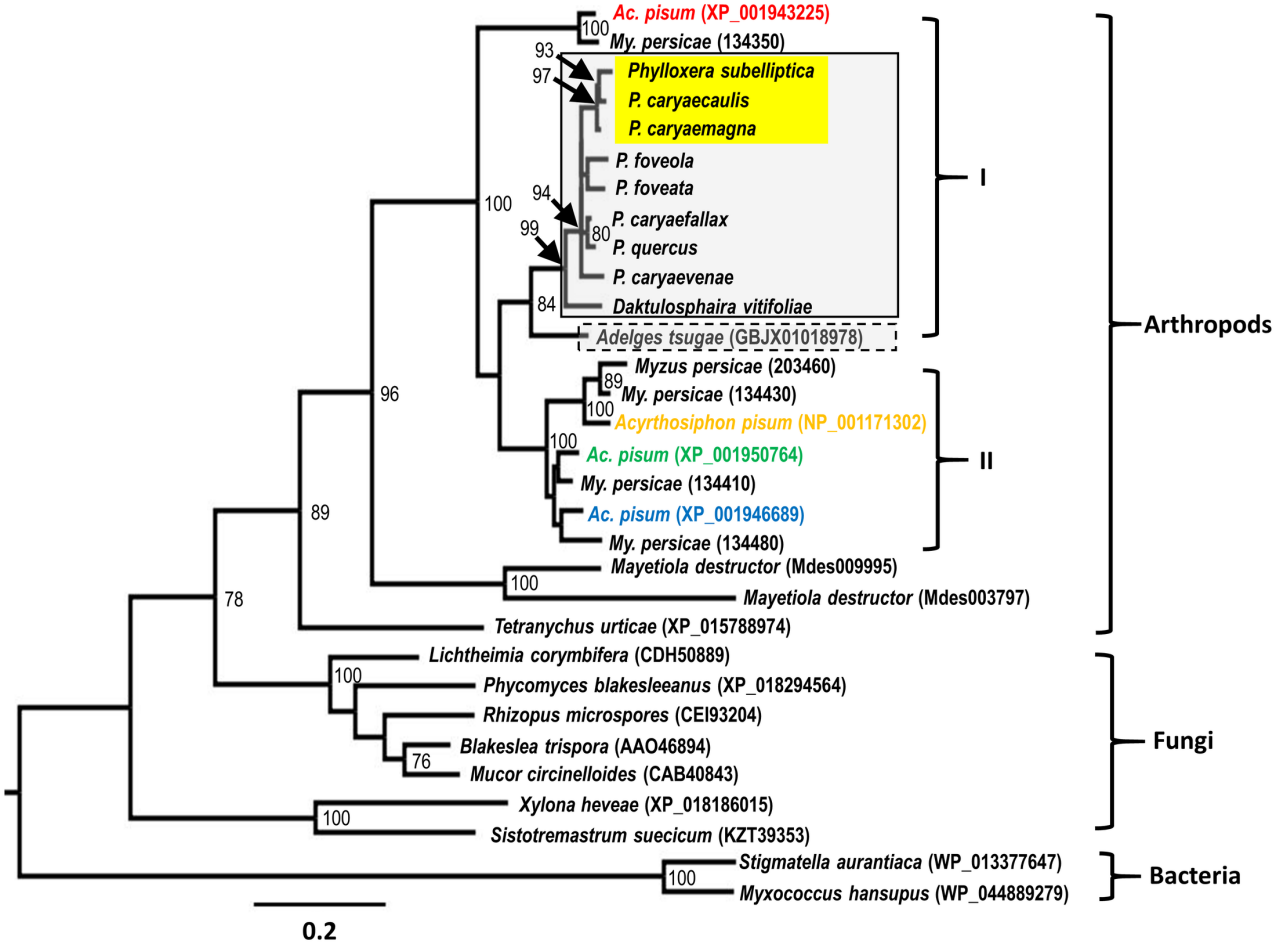
Maximum-likelihood inferred phylogeny of carotenoid desaturase proteins. Accession numbers in parenthesis, insect species boxed and highlighted, *Ac*. *pisum* desaturases in red, blue and green, and other tree labels are indicated as in Fig 3. The *Ac*. *pisum* desaturase NP_001171302 in orange is encoded by a gene that does not form a tail-to-tail pair with a carotenoid cyclase/synthase gene as other *Ac*. *pisum* desaturase genes do in the genome.

We constructed two maximum-likelihood phylogenetic trees using the deduced carotenoid cyclase/synthase and desaturase protein sequences identified in phylloxerids, *Ad*. *tsugae* and *My*. *persicae*, and those previously reported in other arthropod species to infer their relationships. Not surprisingly, Sternorrhyncha species clustered as unique clades closely related to other arthropods and fungi in both trees inferred from cyclase/synthase and desaturase phylogenies, respectively (Figs 3 & 4). The clusters of Sternorrhyncha insects are strongly supported with 100% bootstrap values in both trees, indicating that aphids, adelgids and phylloxerids share common ancestral carotenoid genes that were gained in a single event from a fungal donor through the mechanism of horizontal gene transfer. In agreement with the taxonomic relationship of these three Sternorrhyncha families, both cyclase/synthase and desaturase trees demonstrated that Phylloxerids and Aphids species form their own taxa with adelgids being placed separately. High bootstrap values, 100% for carotenoid cyclase/synthase and 99% for carotenoid desaturase proteins, strongly supported the clustering of Phylloxeridae species. Within the Phylloxeridae clades of both trees, *D*. *vitifoliae* was placed outside of *Phylloxera* species and three stem-gall-inducing species were clustered together as a monophyletic clade with high confidence in spite of the slight positional differences of other leaf-galling, leaf-folding, and free-living *Phylloxera* species (Figs 3 & 4).

In contrast to Phylloxeridae species, each of which appears to have only one cyclase/synthase protein, *Ad*. *tsugae* possesses two, residing within the two Sternorrhyncha cyclase/synthase groups (Group I and Group II, Fig 3), respectively. Group I comprises all Aphididae, Adelgidae, and Phylloxeridae species used and each species contributes a single cyclase/synthase to this group. However, there are no Phylloxeridae proteins within Group II cyclase/synthases but multiple copies of *Ac*. *pisum* and *My*. *persicae* proteins are included, implying the possible gene duplication having occurred in Aphididae. Specifically and supportively, six *My*. *persicae* cyclase/synthase proteins (134360, 134370, 134380–1, 134380–2, 134390 and 134400) whose genes are located in tandem within the genome are clustered in Group II as the closest homologs of *Ac*. *pisum* XP_001950787 (Fig 3).

In the phylogeny of desaturases, Phylloxeridae and Adelgidae species possess single proteins that, together, form a monophyletic group that is separated from Aphididae proteins (Fig 4). In contrast, Aphididae desaturases are present in multiple copies forming four subclades, among which the XP_001943225 (*Ac*. *pisum*)/134350 (*My*. *persicae*) subclade is placed at the base and hence is likely to represent the most ancient one (Fig 4). Three genes encoding *Ac*. *pisum* desaturases (XP_001943225, XP_001946689, XP_001950764) are arranged, respectively, in the tail-to-tail manner with three encoding cyclase/synthases (XP_001943170, XP_003241668, XP_001950787) according to the sequenced *Ac*. *pisum* genome (http://bipaa.genouest.org/is/aphidbase/). Interestingly, the fourth *Ac*. *pisum* desaturase (NP_001171302) that does not pair with a cyclase/synthase gene in the genome was previously demonstrated to confer color polymorphism in aphid populations [13].

## Discussion

In this study, we identified the cartotenoid cyclase/synthase and desaturase genes in nine Phylloxeridae species from their RNAseq assemblies and/or using a genomic DNA PCR approach. These genes are likely to be encoded by the insect genomes and were acquired from a fungal donor through HGT, rather than derived from sequence contaminants of insect endosymbionts, because: 1) nearly all of genes are identified both in insect transcriptomes and from insect genomic DNA preparations; 2) the phylloxerid genes are most closely related to adelgid and aphid genes of fungal origin compared to the microbial homologs; and 3) examination of the *D*. *vitifoliae* genome indicated that these genes are adjacent to those that are highly conserved in insects; for example, the neighboring gene downstream of *D*. *vitifoliae cyclase/synthase* is most similar to insect *transcription initiation factor TIFIID subunit 1* in the NCBI Non-redundant protein database with the top BLASTP (evalue = 0) hit *Ac*. *pisum* XP_003241670 (NCBI accession #).

Although these Phylloxeridae carotenoid genes were horizontally acquired, they seem to be derived from a single HGT event through which single copies of *cyclase/synthase* and *desaturase* were incorporated into the genome of the common ancestor from a fungus, possibly an endosymbiont of the ancestor. This hypothesis is supported by the strong phylogenetic clustering of both genes of these three Sternorrhyncha families, which are separated from homologs of other arthropods and microorganisms (Figs 3 & 4). In addition, carotenoid gene-inferred phylogenetic relationships are consistent to what have been suggested among the three families and within the Phylloxeridae based on previous phylogenetic analysis [30]. Aphididae, Phylloxeridae, and Adelgidae are very closely related, with the latter two families considered to form a superfamily Phylloxeroidea, sister to Aphidoidea [35]. This pattern is also embodied in the phylogeny of carotenoid cyclase/synthase and desaturase genes here. Within Phylloxeridae, both previous studies and this one suggest 1) that some leaf-feeding *Phylloxera* species first switched to feeding on stem tissues and then recolonized stem organs across different hosts as they speciated, and 2) that in a different genus, *D*. *vitifoliae* is separated from all other *Phylloxera* species [30]. The more-recent occurrence of stem-galling *Phylloxera* species is further exemplified in their gaining of an extra 3-nt codon within the second exon of the *cyclase/synthase* coding region, compared to non-stem-galling *Phylloxera* species (Fig 2), a potential molecular marker to distinguish stem-galling from leaf-galling *Phylloxera* spp. These consistencies of evolutionary and phylogenetic relationships, inferred from carotenoid genes and previous studies, respectively, indicated that the carotenoid genes were gained by the common ancestor of aphids, adelgids, and phylloxerids, and later evolved divergently with the differentiation of insect families, genera and species.

In contrast with aphids that possess a variety of numbers of carotenoid genes in their genomes [26], phylloxerids only encode a single copy, for both *cyclase/synthase* and *desaturase* genes. It is likely that aphids experienced multiple lineage-specific gene duplications after their separation from phylloxerids. This is supported by the six *My*. *persicae* cyclase/synthase genes that are clustered in tandem within a genome region, and cluster phylogenetically with the closest relative of the *Ac*. *pisum* gene encoding XP_001950787 (Fig 3). Because *cyclase/synthase* and *desaturase* genes of arthropods are typically arranged to form a tail-to-tail pair, resembling their fungal origin [24], we also examined the arrangement of the two *D*. *vitifoliae* carotenoid genes in the genome and validated that they are likewise within a 25-kb region. Based on the phylogenetic relationships, the number of carotenoid genes in each species, and their positional organization, we hypothesize that phylloxerid carotenoid genes and their closest adelgid and aphid orthologs represent the ancestral forms, and that other carotenoid genes in adelgids and aphids were more recently evolved through gene duplication. Specifically, Group I carotenoid genes evolved prior to Group II as shown in Figs 3 and 4.

Whether and how expansion of carotenoid genes post-HGT in a Sternorrhyncha species are related to life history is an interesting subject. Genome-wide screening of carotenoid genes identified nine *cyclase/synthase* and five *desaturase* genes in *My*. *persicae* in this study compared to three and four, respectively, in *Ac*. *pisum* reported previously [13]. This suggests that *My*. *persicae* experienced selection pressure favoring higher numbers of carotenoid genes possibly to support the generalist herbivore strategy of feeding broadly across hosts [36]. By comparison, *Ac*. *pisum* specializes only on legumes and has fewer carotenoid genes. Phylloxerids are also specialists on few host species but differ in how they feed. Phylloxerids feed on parenchyma, not phloem as adelgids and aphids predominantly do. Compared to other tissues, phloem has higher sugars, sugar alcohols, and osmotic pressure (see [37] and references therein), and transports many defense metabolites [38]. Given that phloem feeders are known to secrete enzymes whose cleavage of plant metabolites may generate free radicals [39], oxidative stress induced during feeding could be quenched by carotenoid scavengers. In contrast, parenchyma-feeding phylloxerids may not suffer from high oxidative stress generated from phloem-feeding and thus may only require low number of carotenoid gene copies.

Recent evidence links carotenoid synthesis in mites to light-regulated diapause and sufficient nutrition [40]. Although the majority of phylloxerids live inside plants, and thereby may be less sensitive to light-regulated diapause, maintaining optimal nutrition likely plays a strong role in phylloxerid evolution. In support of this role in nutrition, the ability to manipulate plant host nutrient availability through galling constrains other insect nutrition genes (e.g., amino acid transporters; [30]) and duplications in carotenoid biosynthesis genes occur among phloem feeding aphidomorphs where nutrients provided by sap are suboptimal. Because one phylloxerid showed no carotenoid desaturase gene expression despite having a complete gene copy, it is possible these genes can be modulated depending on the stress or nutrient environment the insect experiences. It is also possible, however, that the expression of this desaturase gene was not detected due to the RNA sequencing and/or assembly errors. To validate its expression profile, further analysis, *e*.*g*., reverse transcription PCR, should be conducted.

Sternorrhyncha species include plant sap-feeding insects that may have limited access to dietary carotenoids [29]. Within this suborder, Phylloxeridae is the third family that encodes insect-derived carotenoid genes. Previous attempts of identifying carotenoid genes failed to amplify a fragment from *Bemisia tabaci* (Family Aleyrodidae) and *Pachypsylla venusta* (Family Psyllidae) using PCR, and failed to obtain a sequence hit from *Diaphorina citri* (Family Psyllidae) EST database [26]. Using the carotenoid gene identification protocol described in the ‘Materials and Methods’ above, *i*.*e*., TBLASTN searching *Ac*. *pisum* carotenoid proteins followed by NCBI non-redundant protein database validation, we were unable to find carotenoid genes in the genome assemblies of *B*. *tabaci* (NCBI # ASM185493v1) and *Dactylopius coccus* (Family Coccidae, NCBI # GCA_000833685.1). These data suggest that Sternorrhyncha-derived carotenoid genes may only be restricted to Aphididae, Adelgidae, and Phylloxeridae. Intriguingly, *B*. *tabaci* harbors an obligate bacterial endosymbiont *Portiera* that synthesizes carotenoids for its host as a different mechanism [29].

In conclusion, phylloxerids retain carotenoid genes with the same origin as those in aphids and adelgids: horizontally transferred from a fungus. These genes did not duplicate but are maintained as single copies in the genomes of phylloxerids, specialist species feeding on parenchyma rather than plant sap. This study provides insight into the evolution of Phylloxeridae species, mechanisms underlying their nutritional adaptation, and insight into the role of carotenoid biosynthesis in animals.

## Supporting information

S1 File Carotenoid cyclase/synthase coding sequences identified from transcriptomes of nine Phylloxeridae species. (TXT)

S2 File Carotenoid desaturase coding sequences identified from transcriptomes of nine Phylloxeridae species. (TXT)

S3 File Degenerated PCR primers used to amplify carotenoid genes from Phylloxeridae genomic DNA. (DOCX)

S4 File Cyclase-synthase fusion proteins used for phylogenetic analysis. (TXT)

S5 File Desaturase proteins used for phylogenetic analysis. (TXT)
