# Supplementary material for: Phylloxerids share ancestral carotenoid biosynthesis genes of fungal origin with aphids and adelgids
Source: PLoS One. 2017 Oct 11;12(10):e0185484. doi: 10.1371/journal.pone.0185484 (PMC5636080; doi:10.1371/journal.pone.0185484)
Supplement: S3 File — (DOCX) [file pone.0185484.s003.docx]

**S3. Degenerated PCR primers used to amplify carotenoid genes from Phylloxeridae genomic DNA.**

| **Gene** | **Frag.** | **First-round PCR Primers** | **Second-round PCR Primers** |
| --- | --- | --- | --- |
| *Carotenoid synthase/cyclase* | *cs-1* | cs1f1:  RRYGTYCATCTTAYTTACAC  cs1r1:  TCTGAYTTTCGRYCCGCAAA | cs1f2:  GTTRRCAYTAATAACATGGC  cs1r2:  TCRATCATATCGTCAGTAAC |
| *Carotenoid synthase/cyclase* | *cs-2* | cs2f1:  TCKTTYAAYTTGGCCAGCTT  cs2r1:  GTAATTSYRACTAGRATTTT | cs2f2:  THCGATTRGACYTGATGATT  cs2r2:  TTAAGRGAAGCTCTTTTAGG |
| *Carotenoid desaturase* |  | ds-f1:  GATTAAGTAAACAAGGATTTCA  ds-r1:  AACACCAGTACCAGGTTG | ds-f2:  GYCATCGTTTTGATCAAGG  ds-r2:  TAGATTAAATTCTTTTTGCCA |
